# Is computerised CBT really helpful for adult depression?-A meta-analytic re-evaluation of CCBT for adult depression in terms of clinical implementation and methodological validity

**DOI:** 10.1186/1471-244X-13-113

**Published:** 2013-04-15

**Authors:** Mirai So, Sosei Yamaguchi, Sora Hashimoto, Mitsuhiro Sado, Toshi A Furukawa, Paul McCrone

**Affiliations:** 1 Department of Psychological Medicine, Institute of Psychiatry, Kings College London, UK, Weston Education Centre, Cutcombe Rd, London, SE5 9RJ, UK; 2 Department of Psychiatric Rehabilitation, National Institute of Mental Health, National Centre of Neurology and Psychiatry, Japan, 4-1-1 Ogawa-Higashi, Kodaira, Tokyo, 187-8553, Japan; 3 Department of Psychology and Humanities, College of Sociology, Edogawa University, 474 Komaki, Nagareyama, Chiba, 270-0198, Japan; 4 Department of Neuropsychiatry, Keio University School of Medicine, 35 Shinanomachi, Shinjuku-ku, Tokyo, 160-8582, Japan; 5 Department of Health Promotion and Human Behavior/Department of Clinical Epidemiology, Kyoto University Graduate School of Medicine/School of Public Health, Yoshida Konoe-cho, Sakyo-ku, Kyoto, 606-8501, Japan; 6 Centre for the Economics of Mental and Physical Health (CEMPH) P024, Institute of Psychiatry King’s College London, De Crespigny Park, London, SE5 8AF, UK

**Keywords:** CCBT, Web-based CBT, Cognitive-behavioural therapy, Depression, Systematic review, Meta-analysis

## Abstract

**Background:**

Depression is a major cause of disability worldwide, and computerised cognitive behavioural therapy (CCBT) is expected to be a more augmentative and efficient treatment. According to previous meta-analyses of CCBT, there is a need for a meta-analytic revaluation of the short-term effectiveness of this therapy and for an evaluation of its long-term effects, functional improvement and dropout.

**Methods:**

Five databases were used (MEDLINE, PsycINFO, EMBASE, CENTRAL and CiNii). We included all RCTs with proper concealment and blinding of outcome assessment for the clinical effectiveness of CCBT in adults (aged 18 and over) with depression. Using Cohen’s method, the standard mean difference (SMD) for the overall pooled effects across the included studies was estimated with a random effect model. The main outcome measure and the relative risk of dropout were included in the meta-analysis.

**Results:**

Fourteen trials met the inclusion criteria, and sixteen comparisons from these were used for the largest meta-analysis ever. All research used appropriate random sequence generation and Intention-to-Treat analyses (ITT), and employed self-reported measures as the primary outcome. For the sixteen comparisons (2807 participants) comparing CCBT and control conditions, the pooled SMD was −0.48 [95% IC −0.63 to −0.33], suggesting similar effect to the past reviews. Also, there was no significant clinical effect at long follow-up and no improvement of function found. Furthermore, a significantly higher drop-out rate was found for CCBT than for controls. When including studies without BDI as a rating scale and with only modern imputation as sensitivity analysis, the pooled SMD remained significant despite the reduction from a moderate to a small effect. Significant publication bias was found in a funnel plot and on two tests (Begg’s p = 0.09; Egger’s p = 0.01). Using a trim and fill analysis, the SMD was −0.32 [95% CI −0.49 to −0.16].

**Conclusion:**

Despite a short-term reduction in depression at post-treatment, the effect at long follow-up and the function improvement were not significant, with significantly high drop-out. Considering the risk of bias, our meta-analysis implied that the clinical usefulness of current CCBT for adult depression may need to be re-considered downwards in terms of practical implementation and methodological validity.

## Background

Depression is recognised as a major cause of disability all over the world [1]. It has also been recently emphasised that it is not a highly recoverable disorder, even when treated with established pharmacotherapy [2], challenging previously widely-held ideas on its treatability [3]. Therefore, there is a great amount of expectation placed on evidence-based cognitive-behavioural therapy (CBT) [4] as an alternative or addition to pharmacotherapy [5,6]. It is hypothesised that reconstructing distorted cognition or inadaptable behaviour with CBT is likely to lead to the reduction of symptoms [7]. In fact, there has been increasing attention paid to CBT because of several of its advantages, including its significant effectiveness against mild-to-moderate depression [8], the enhancement of quality of life [9], increase in adherence to pharmacotherapy [3], comparative advantage for pregnant women [10] and patients’ preferences [11]. Also, CBT seems to be beneficial as an early intervention or relapse prevention measure [12] and CBT is recommended by the National Institute for Health and Clinical Excellence (NICE) in preference over routine pharmacotherapy as a treatment for milder depression [13]. Nevertheless, making CBT widely used requires addressing inevitable resource allocation problems, including accessibility and cost-effectiveness [14]. Therefore, the advantages and practicality of self-help treatments including computerised CBT (CCBT), i.e. self-help CBT using a programme on a website or on a computer without an online network, have been attractive, and it is believed that self-help CBT will be an efficacious intervention, especially for mild-to-moderate depression [15]. There are now even greater expectations of CCBT, owing to its increased potential due to technological progress in terms of interactivity, multimedia functions and flexibility [16]. Since the first randomised controlled trial (RCT) reported by Selmi in 1990 [17], the number of papers published on CCBT has increased markedly. Also, to date, there have been five meta-analyses [18-22] which specifically looked at the effect of CCBT on adult depression, and all of them found that CCBT was of benefit with moderate effect sizes.

However, these systematic reviews cannot be considered to provide definitive and compelling support for CCBT due to both the lack of two significant perspectives from clinical implementation and the four issues of methodological validity.

From the perspective of clinical implementation, one point is that they have never dealt with functionality. Indeed, all of the three RCTs carried out to date on the cost-utility of CCBT [23-25] could not find significant QALY increases due to the lack of functional improvement. In addition to functionality, on the other hand, it seems to be also insufficient that even long-term effectiveness has not been meta-analysed at all.

Considering methodological aspects, it seems that past meta-analyses have crucial limitations in quality. The first issue is the heterogeneity of both targeted disorders and intervention. In the former, three meta-analytic studies [18,19,21] dealt with depression and other disorders (such as anxiety disorders) as common target disorders, implying a critical bias in the results due to considerable diagnostic heterogeneity, as CBT for depression and that for other disorders, such as anxiety disorders, are theoretically different interventions. Indeed, in these three, the effect sizes of CCBT for anxiety were generally greater than CCBT for depression.

In contrast, two other meta-analyses [22,20] exclusively dealt with depression. Nevertheless, there still remain the heterogeneous problems of intervention. Gellatly et al. included not only ten CCBT research studies but also twenty nine studies with non-CCBT interventions, such as bibliotherapy, indicating that the results of this meta-analysis cannot be accepted as those of solely CCBT-intervention. Compared to this review, Andrews et al. used the largest number to date (eleven) of RCTs on depression-only CCBT. Even so, this meta-analysis included two inappropriate studies [17,26], as mentioned below under Results.

Secondly, the published systematic reviews have not paid due attention to the problem of dropouts. Some studies suggest that CCBT has higher attrition rates than other therapies. However, one meta-analysis by Waller and Gilbody [27] indicated that there was no significant difference between CCBT users and controls, but this meta-analysis had substantial diagnostic heterogeneity (two depression-specific studies and seven studies specific to other disorders). Since uneven attrition between or among intervention arms can be a significant cause of bias [28], more rigorous consideration needs to be given to this factor.

Thirdly, the published meta-analyses have not examined publication bias in the available literature on CCBT.

Lastly, there have been an increasing number of new studies published since the most recent systematic review.

Therefore, we conducted a meta-analysis of clinical effectiveness of single CCBT for adult depression, taking the above methodological factors into consideration with an additional evaluation of functional outcomes and long-term follow-up effects.

## Methods

### Identification and selection of studies

All RCTs completed and analysed by 11 July, 2011 were eligible for inclusion in this review. Five bibliographic databases were used [MEDLINE (1948 to July 2011), PsycINFO (1806 to July 2011), EMBASE (1980 to 2011), CENTRAL (Cochrane Library, 2011 latest issue), and CiNii (until July 2011)]. We also searched http://www.controlled-trials.com. Multiple search terms were used (Appendix) and modified for each database, as necessary. The search was performed on 11 July, 2011.

We included 1) randomised trials 2) in which the effects of guided and unguided CCBT specific to depression 3) were compared with one or more control conditions 4) in individuals aged 18 years or older 5) with depression, and in which 6) reliable and standardised rating-scales were equally used both at baseline and follow-up. Also, we only included studies 7) with proper allocation, concealment, and single or greater blinding of outcome assessment; and 8) trials using medications or other psychotherapies were included. We excluded studies on 1) inpatients, because we excluded patients with severe symptoms from self-help intervention, and those with 2) comorbidities such as psychotic disorders, manic status, dementia and severe physical conditions. In fact, we had originally intended to distinguish between patients on waitlists from treatment as usual (TAU), because we considered there to be restrictions on administration of medications to patients on waitlists. Nevertheless, the proportion of subjects taking medication at waitlist baseline was very similar to that with TAU, and medication was mostly not controlled. Therefore, we decided to group together both of these, and checked the influence of this factor on outcomes through a subgroup analysis. This grouping seemed to be justified because the above past five meta-analyses had treated data likewise. Studies had to have a primary endpoint including a measure of depression at the outcome assessment immediately after intervention and at long follow-up (if applicable). We defined long follow-up as follow-up where the final assessment was more than six months after treatment, because this is a recovery period associated with low future recurrence of depression [29]. Function at post-treatment and the number of total dropouts were adopted as secondary endpoints.

### Meta-analyses

Intervention effects were expressed using various types of rating scales for common outcomes, thus the effect sizes using standardized mean differences (SMDs) with 95% confidence intervals for post-treatment were computed, and then incorporated into the meta-analysis and presented with 95% confidence intervals. Where trials used a number of different tools to assess depression, we included the main outcome measure following our hierarchy, including the primary endpoint or endpoint first reported in the results.

Statistical heterogeneity was evaluated through a SMD forest plot. Cochrane’s Q statistic (chi-squared test) was performed with a significance level of 0.10. Furthermore, the I-squared (I [2]) statistic for heterogeneity was also used for confirmation of Cochrane’s Q statistic. A random-effect model was selected due to the large heterogeneity of each clinical design and participants. All meta-analyses were performed using Review Manager (RevMan ver. 5.1). Subgroup analyses were performed for the type of control (Waitlist and TAU). Also, we re-evaluated the clinical effectiveness through a sensitivity analysis by excluding Beck Depression Inventory I, BDI- I [30], and II [31], or according to the difference in attrition rates and imputation techniques. The reason for the former is that, particularly in CCBT studies primarily relying on self-rating scales, measurement bias is suspected due to differences between the scales employed, and it is necessary to avoid this giving rise to underestimation. The reason for the latter is that high dropout rates were expected [27], thus we also performed an analysis excluding research with attrition rates higher than 20% (such rates probably had an influence on the results irrespective of ITT according to Cochran handbook) [32], significantly higher dropout RRs or non-modern imputation processing.

A funnel plot was used as a test of the main outcome to detect publication or reporting bias through visual inspection. Begg’s [33] and Egger’s [34] tests were also conducted for statistical checking. When a significant small study effect was noted, we assessed its influence through the trim-and-fill method [35].

It seems that adequate missing value management is useful in carrying out appropriate CCBT evaluation, because we expect overall attrition rates to be high. Although it was physically unfeasible for us to collect all the original data without imputation, in the present meta-analysis we considered the potential impact on the review result through a sensitivity analysis in terms of the influence of imputation. Also, the modern imputation was defined as an imputation needing more complex processing than classic and comparatively simple imputations such as last observation carried forward (LOCF) or mean imputation (MI).

## Results

### Characteristics of included studies

**Figure 1:**
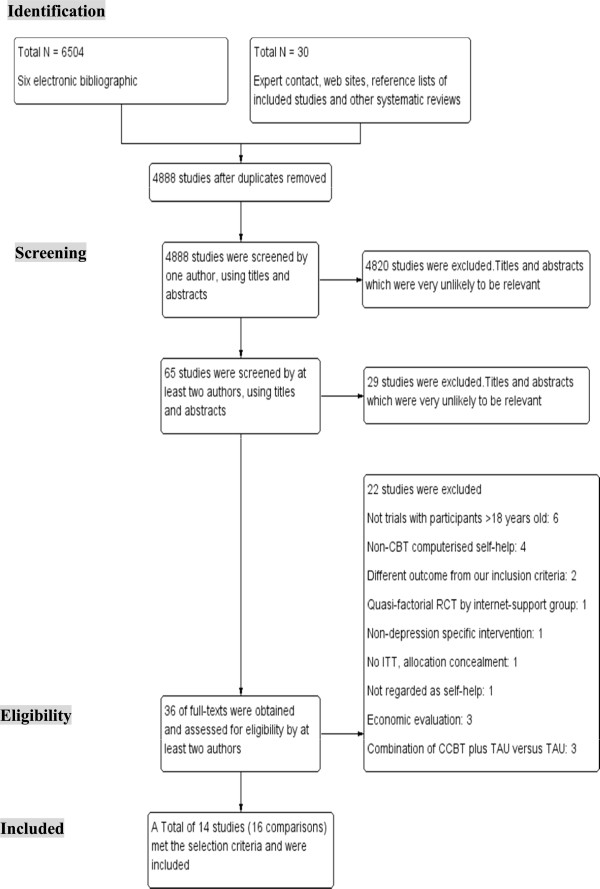
Process of study selection: PRISMA flow diagram.

Out of 4,888 studies initially screened, following the process shown in Figure 1, fourteen were identified as relevant to the investigation of the clinical-effectiveness of CCBT as the sole intervention (Table 1). All continuous mean and SD values of post-treatment primary outcome data were available, and data from all 14 were finally used for 16 comparisons in the meta-analysis, as mentioned below (Table 2). In this inclusion process, we excluded two significant studies by Selmi et al. [17] and Wright et al. [26] that were included in all of the five prior meta-analyses mentioned above, because we ultimately judged one trial by Selmi et al. to have been conducted without an ITT analysis, proper allocation concealment and random sequence generation. The other, by Wright et al., was not regarded as being a study on self-help, due to highly intensive assistance of eight standard CBT sessions.

**Table 1:** Selected characteristics of randomised controlled studies examining the effect s of CCBT for adult depression

**First author and year of publication**	**Rrecruitent**	**Intervention, number of modules**	**N**	**Outcome measures**	**Function measures**	**Control group**	**F-U**	**Attrition rate (%)**	**Imputation**	**Country**
Andersson (2005) [36]	community	guided iCBT, 5	117	BDI, MADRS	QOL	WL (online discussion group)	6 months	27%	LOCF	Sweden
Christensen (2004) [37]	community	unguided iCBT, 5	525	CES-D	na	Attention placebo	6 weeks	20%	LOCF	Australia
Clarke (2002) [38]	vio HMO	unguided iCBT, 7	299	CES-D	na	TAU	8 months	41%	unclear	USA
Clarke (2005)a	vio HMO	unguided iCBT, 7 + postcard-reminder	175	CES-D	SF-12	TAU	16 weeks	34%	REML	USA
Clarke (2005)b	vio HMO	unguided iCBT, 7 + telepone-reminder	180	CES-D	SF-12	TAU	16 weeks	34%	REML	USA
Clarke (2009) [39]	vio HMO	unguided iCBT, 4	160	PHQ-8	na	TAU	8 months	37%	REML	USA
de Graaf (2009) [40]	community	unguided iCBT, 8 or 9	303	BDI-ii	SF-36	TAU	6 months	5%	MI	the Netherlands
Meyer (2009) [41]	community	guided iCBT, 8	396	BDI	WSAS	WL	6 months	31%	replaced by pretreatment scores	German
Perini (2009) [42]	community	guided iCBT, 8	45	PHQ-9, BDI-ii	SDS	WL	8 weeks	27%	replaced by pretreatment scores	Australia
Ruwaard (2009) [43]	community	guided iCBT, 11Weeks	54	BDI	WBQ	WL	18 months	9%	WOCF	the Netherlands
Spek (2007) [44]	community	unguide iCBT, 8	301	BDI-ii, EDS	na	WL	2 weeks	40%	MI	the Netherlands
Titov (2009)a	community	technician-guided iCBT, 8	81	PHQ-9, BDI-ii	SDS	WL	16 weeks	8%	LOCF	Australia
Titov (2009)b	community	clinician-guided iCBT, 8	86	PHQ-9, BDI-ii	WBQ	WL	16 weeks	8%	LOCF	Australia
van Straten (2008) [45]	community	guided iCBT, 4	213	CESD, MDI	EuroQoL	WL	4 weeks	17%	MI	the Netherlands
Venrnmark (2010) [46]	community	guided iCBT, 8	88	BDI, MADRS	QOL	WL	6 months	14%	LOCF	Sweden
Warmerdam (2008) [47]	community	guided iCBT, 8	263	CESD	EuroQoL	WL	12 weeks	40%	LMM	the Netherlands

*iCBT*, Internet-based Cognitive Behavioural Therapy; *cCBT*, Computer-based Cognitive Behavioural Therapy; *BDI*, Beck Depression Inventory; *MADRS*, Montgomery Asberg Depression Rating Scale; *HADS*, Hospital Anxiety and Depression Scale; *CESD*, Centre for Epidemiological Studies Depression Scale; *PHQ-9*, Patient Health Questionnaire 9-item; *EDS*, Edinburgh Depression Scale; *MDI*; Major Depression Inventory; *QOL*, Quality of Life Inventory; *WSAS*, Work and Social Adjustment Scale; SF-12(36), Short Form 12(36) Health Survey; *WBQ*, Well-Being Questionnaire; *SDS*, Sheehan Disability Schedule; *WL*, Waitlist; *TAU*, Treatment as Usual; *LOCF*, Last Observation Carry-forward; *REML*, Residual Maximum Likelihood Estimation; *WOCF*, Worst Observation Carry-forward; *MI*, Mean Imputation; *LMM*, Linear Mixed Modelling.

**Table 2:** Data and analyses

**Comparison 1. CBT versus control**				
**Outcome or subgroup**	**No. of comparisons**	**No. of participants**	**Statistical method**	**Effect estimate**
1 Reduction in depression symptoms post treatment	16	2807	Std. Mean Difference (IV, Random, 95% CI)	−0.48 [−0.63. -0.33]
2 Reduction in depression symptoms at long follow-up	5	976	Std. Mean Difference (IV, Random, 95% CI)	−0.05 [−0.19, 0.09]
3 Improvement in function at post-treatment	13	2008	Std. Mean Difference (IV, Random, 95% CI)	−0.05 [−0.31, 0.22]
4 Relative risk of attrition at post-treatment	16	2807	Risk Ratio (M-H, Random, 95% CI)	1.68 [1.31, 2.16]

There were more women than men subjects, and the mean age ranged from 22.6 years (Clarke 2009) to 55 years [44] (see the Characteristics of included studies table). The majority of CCBT programmes were based on standard CBT, while combined CBT with other therapies was used in two trials. All studies used a self-reported measure of depression as their primary outcome, and eight trials mainly used BDI. All studies stated that allocation concealment and ITTs were adequately performed. There were follow-up data beyond six months after the interventions for 7 trials, one study (Titov 2009) [48] finished the follow-up of only the intervention group, and thus control data were not available. Another study [43] presented the results of the follow-up at 18 months by transforming a waiting-list group into an intervention group after a first-phase trial, and therefore could not be included in the current meta-analysis. Ten clinical-effectiveness studies recorded functional change as well as mood. Also, all 14 studies showed the post-intervention attrition rate. Two studies (Clarke 2005 [49] and Titov 2009 [48]) that each had three branches in the original research were respectively divided into two comparisons, and they were identified as Clarke 2005a and Clarke 2005b (post-card reminder or telephone reminder with CCBT) in the former, and as Titov 2009a and Titov 2009b (either technician-assisted or clinician-assisted CCBT) in the later, thereby giving a total of 16 comparisons from 14 original studies for the meta-analysis.

### Primary and secondary outcomes

**Figure 2:**
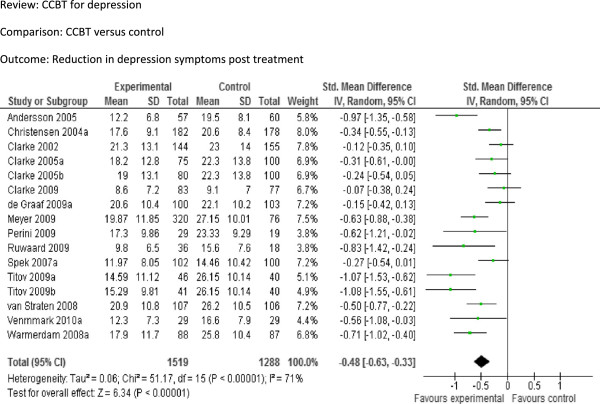
Comparison: CCBT versus control, Outcome: Reduction in depression symptoms at post-treatment.

**Figure 3:**
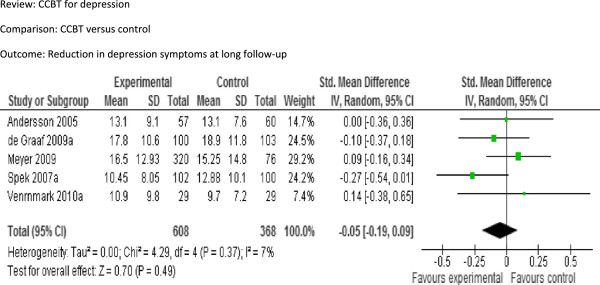
Comparison: CCBT versus control, Outcome: Reduction in depression symptoms at long follow-up.

**Figure 4:**
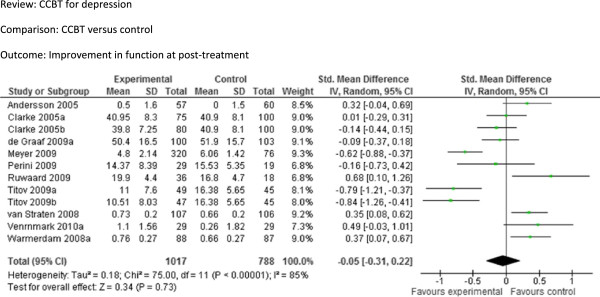
Comparison: CCBT versus control, Outcome: Improvement in function at post-treatment.

**Figure 5:**
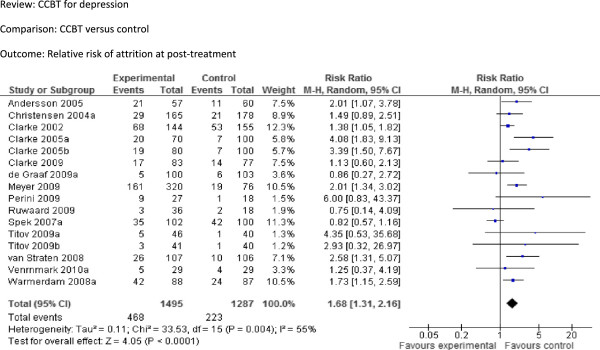
Comparison: CCBT versus control, Outcome: Relative risk of attrition at post-treatment.

We included 16 comparisons (2807 participants) in terms of the reduction in depression symptoms between CCBT and controls following treatment (Figure 2). The pooled SMD was −0.48 [95% CI −0.63 to −0.33], indicating a significant moderate effect. Reduction in depression symptoms at long follow-up (Figure 3): The pooled SMD from the five studies (976 participants) that provided long-term follow-up data was −0.05 [95% CI −0.19 to 0.09], indicating no significant difference between the groups. Improvement in function at post-treatment (Figure 4): The pooled SMD from the twelve comparisons that provided data on function at post-treatment was −0.05 [95% CI −0.31 to 0.22], indicating no significant difference between two groups. In addition, the pooled relative risk from all trials providing data on attrition at post-treatment (Figure 5) was 1.68 [95% CI 1.31 to 2.16], indicating a significant difference between the two groups.

### Subgroup analyses

**Figure 6:**
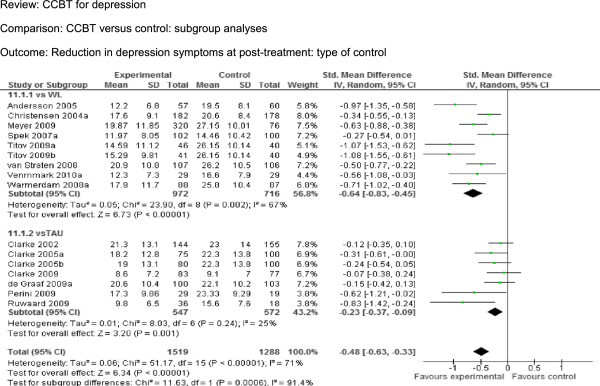
CCBT versus control: subgroup analyses – type of control (Waitlist and TAU), Outcome: Reduction in depression symptoms at post-treatment.

We attempted to analyse the differences in effects between studies where the control was a waiting list and those where it was TAU (Figure 6). The control was a waitlist in nine comparisons [36,37,41,44-48], while it was TAU in seven comparisons [38-40,42,43,49]. The pooled SMD for waitlist-controlled trials was −0.63 [95% CI −0.83 to −0.45], indicating a moderate effect. By contrast, the pooled SMD for TAU-controlled trials was −0.23 [95% CI −0.37 to −0.09], indicating a small effect. There was a significant difference between two groups.

### Sensitivity analyses

Sensitivity analyses were conducted as show below.

### Rating scales except BDI

For the seven comparisons that employed neither BDI -I nor -II as the primary outcome measure [37-39,45,47,49], the SMD was −0.32 [95% CI −0.48 to −0.17], indicating a small clinical effect in favour of CCBT.

### Acceptable attrition rate (<20%)

For the seven comparisons that were reported to have an acceptable dropout rate (<20%) [37,40,43,45,46,48], the SMD was −0.59 [−0.85, -0.34], showing a moderate clinical effect in favour of CCBT.

### Imputation techniques

For the nine comparisons without a significant difference in attrition rate between intervention and control at post-treatment [37,39,40,42-44,46,48], the SMD was −0.50 [95% CI −0.73 to −0.27], showing a moderate clinical effect in favour of CCBT. On the other hand, for the eight comparisons from seven trials with modern imputation techniques [38,39], [43-45,47,49], the SMD was −0.34 [95% CI −0.51 to −0.18], indicating a small clinical effect (of borderline significance) in favour of CCBT.

### Publication bias

**Figure 7:**
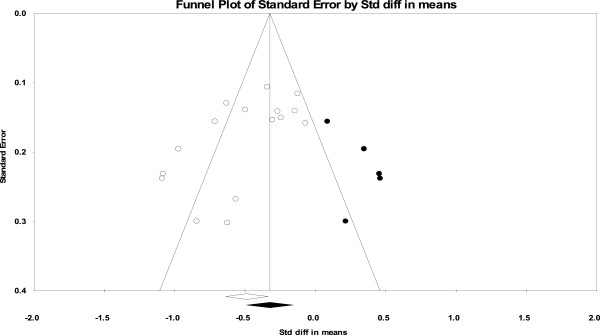
**Funnel plot of comparison: CCBT versus control, outcome: reduction in depression symptoms at post-treatment.** White circles: included comparisons. Black circles: imputed comparisons using the trim-and-fill methods. White diamond: pooled observed standard mean difference. Black diamond: pooled imputed standard mean difference.

We explored publication bias, using a Funnel plot (Figure 7). The plot can be seen to be asymmetric, indicating a relationship between intervention effect and study design. In particular, this asymmetry suggests a publication bias toward larger effect size in smaller studies, since there was a marked concentration of studies shown on the left side in the lower part of the plot. It was inferred that smaller studies with larger effect sizes were more likely to be published, and thus they had a higher probability of demonstrating statistical significance. Also, there was significant statistical evidence for study bias using both Begg’s test (p = 0.009) and Egger’s test (p = 0.01). Using the trim and fill analysis, the SMD was −0.32 [95% CI −0.49 to −0.16].

## Discussion

We analysed 16 comparisons from 14 publications, targeting the largest size and highest quality meta-analysis. Consequently, this review found that CCBT has apparently a moderate post-treatment effect size (SMD −0.48, 95% CI −0.63 to −0.33) for adult depressive symptoms compared with control conditions, indicating almost the same result as those of past meta-analyses. Nevertheless, we further found a possibility that this result may have to be re-considered downward in terms of practical implementation and research methodology. Thus, we would like to examine these two aspects.

Considering the lack of the endurance of effectiveness, functional improvement and the high dropout rate, our result inevitably casts doubt on the actual practicability of the current CCBT for depression.

To begin with, the attenuation of long-term effectiveness with CCBT seems to be a serious issue from a clinical point of view. In fact, although one of the past reviewers [20] implied this tendency, long follow-up has not been clearly reviewed in meta-analyses until today. In this context, ours is the first review to meta-analyse long follow-up outcomes. This was paradoxical from the perspective that it has been reported that the effect of standard face-to-face CBT on depression does not usually attenuate sharply after intervention even without maintenance sessions. For example, the latter view has been advocated by the Annual Review of Psychology [50], which mentions that the effectiveness of CBT appears to be at least more enduring than that of antidepressants for depressive patients. However, it is still unclear why such variance could arise according to differences in modality, while van Londen et al. raised this question in the context of bibliotherapy [51].

In addition to long follow-up outcomes, it also has not been meta-analysed until our study about whether CCBT can contribute to functional improvement, even though this outcome is critically important in view of evaluating cost-utility, which is referred to as a distinctive advantage of CCBT [15]. In our analysis, CCBT intervention did not provide a significant effect in terms of function. There are a few possible reasons for this. Firstly, current CCBT may not be fundamentally good enough to improve function. The attainment of social functions such as returning to work has been commonly recognised as being more difficult than simply reducing depressive symptoms [52]. Secondly, we may have to consider the scale-sensitivity of function. Revicki et al. also referred to the property of generic measures that improvements in those scores are less sensitive in less depressive patients [53]. They suggested that such generic scales were even more unlikely to change amongst mild-to-moderate depressive patients than in severe depressive patients, often resulting in little change in utility and problematic utility assessment.

The third issue with practical implementation is that more than half of included studies had high overall dropout rates. It is clear that the higher dropout is unavoidable, especially for depression remediation, in that poor motivation is one of the fundamental symptoms. Indeed, even in the NHS, the dropout rate from CCBT is also high, with up to 50% of users starting the programme for depression not completing it, and it seems that this needs to be addressed as a serious issue [54].

Despite the above substantial limitations of CCBT, it is still used on the premise that it is significantly effective, at least as measured immediately following treatment with it. However, by addressing methodological issues, our analysis further revealed some findings that may raise a more fundamental question of whether CCBT is really effective for adult depression even following treatment.

The first finding is the ambiguous definition of control conditions. In all previous systemic reviews of CCBT, there was little clarification of the influence of grouping results from studies with TAU and the waitlist as controls. Unlike research on medications or psychotherapy, all RCTs on CCBT effectively did not restrict the usage of medications for waitlist groups. Therefore, we had held that this confusion between groups without sufficient presentation is a considerable problem, and set up a protocol to separate subjects on waitlists from those undergoing TAU. However, we found that the proportion of patients taking medication at baseline for TAU groups was in the range from 0% to 76%, and the range for control groups was from 37% to 74%. When considering the virtually undistinguishable rates of medication intake, we concluded that it was difficult to clearly separate TAU from waitlist data, and that is why we classified TAU and waitlist subjects into the same control group in a post-hoc decision, adding a subgroup analysis on the influence of doing this.

In the subgroup analysis, our results showed that the effects were significantly greater when the control group was a waitlist as opposed to TAU. Only a meta-regression [20] had an identical finding to ours, although the analysis was conducted by using only four (10.2%) reliable studies with depression-specific CCBT intervention. In general, this type of difference seems rational because TAU is more therapeutically intensive than a waitlist. However, another likely cause is that the reason for this is due to the tendency for it to be fundamentally easier for an intervention group to indicate a greater effect size relative to a waitlist than active placebo in psychotherapy research [55]. Therefore, it has been recently recommended to not use waitlists in research designs because of overestimation of intervention. Either way, this issue should be treated more carefully in general RCT settings as well as in RCTs of CCBT.

The second issue was that a high attrition rate was also considered to lead to a significant bias despite the conduct of ITT throughout all included studies. In practice, Cochran states that attrition rates higher than 20% may even affect outcomes analysed using ITT [32]. Also, extremely uneven attrition between or among branches of intervention can be an impermissible cause of bias [28]. Only one meta-analysis by Waller and Gilbody has dealt with this attrition issue, finding that subjects treated with CCBT dropped out approximately twice as frequently as control subjects, but this finding was not statistically significant [27].

In relation to the high dropout rate, we focused on the fact that a variety of imputation techniques were implemented for ITT in order to cover attrition in each study, but there was no research on CCBT which examined this risk by this kind of imputation. Rickels and Schweizer mentioned that ITT takes account of dropouts, usually by LOCF [56]. However, Shao et al. [57] and Unnebrink et al. [58] claim that old-type imputations, such as LOCF, mean imputation and worst observation carry-forward (WOCF), can cause significant differences in results when the attrition rate is higher than 20%. By contrast, modern imputation can be thought as being more appropriate. Moreover, there can be significant differences even among imputations, and if so, this issue is serious for research especially where there is a high level of attrition. For example, Warmerdam demonstrated that newer imputation led to significantly different results [25]. Therefore, we investigated the probability of bias due to the method of imputation. In fact, when only trials with modern imputation techniques were included, the effect size decreased from moderate to mild. The influence of imputation has not been seriously discussed in psychotherapy, including self-help. In particular, research on CCBT should give more consideration to this because of its high attrition rate relative to other psychotherapies.

Thirdly, our study was the first to detect significant publication bias specific to CCBT, and this suggested the necessity of careful re-consideration in evaluating the usefulness of CCBT. Indeed, the trim-and-fill method suggested that the SMD reduced from −0.48 [95% CI −0.63 to −0.33] to −0.32 [95% CI −0.49 to −0.16], but still indicating significant effectiveness at least at post-treatment.

Finally, we cannot overlook the fact that there has been a remarkable dominance of self-rating scales used as the primary endpoints of past CCBT research. In our analysis, self-rating scales were used as the primary outcome in all studies. Although all the adopted scales were academically reliable as screening tools, excessive expectations as to self-rating measures could lead to significant bias in the results because self-report ratings from depressed patients are not necessarily a reliable or definitive estimate of the severity especially during the acute phase including before symptomatological improvement [59].

Our sensitivity analysis also demonstrated that the effect size at post-treatment reduced from moderate to small without BDI. This can be explained by the characteristic of BDI that the score tends to be significantly influenced by cognitive factors rather than other instrumentals due to the different conceptualisation of depression among scales [60,61]. Indeed, CCBT is more likely to improve BDI scores than other measures probably because CCBT programmes strategically target cognitive change. Further, it has been also discussed that BDI is inaccurate as a way of appraising treatment outcomes due to overreactivity [62,63]. The frequent use of BDI can be theoretically justified as an efficacy study aiming at the efficacious maximisation of intervention. Even so, in terms of generalisablity, we may need to keep in mind the risk of overestimation when using self-rating scales, including BDI, when actually adopting CCBT for clinical use.

It is seemingly reasonable to expect that self-help CCBT can be a clinically- and cost-effective intervention, considering prior wholly-supportive reviews; however, the use of CCBT, even for mild to moderate depression, may be less practical and efficacious than believed at present. This can be supported by the poor results of three available cost-utility analyses of depression CCBT [23-25]. Nevertheless, it would be too extreme to conclude that CCBT is an inefficacious intervention for adult depression for a few reasons. Firstly, we could distinguish indications for which CCBT is appropriate. In fact, it has been reported that applying CCBT to patients with a personality suitable for it [64] or to those from a technologically-literate generation [19] may contribute to better outcomes. Also, further development of CCBT in terms of sophistication and attractiveness accompanying the rapid progress of information technology [65] might enhance the effectiveness of and adherence to CCBT, such as in the format of a therapeutic computer game [66].

Our review has a few limitations. Firstly, we should have ideally recalculated the effect size (SMD) of each outcome from the original research data in order to enhance the review quality [32]. However, we could not do this due to physical and time restrictions. Secondly, we could not include unpublished data or data from on-going trials even though we attempted to collect them using several ways.

## Conclusion

This review found that CCBT seems to improve depressive symptoms at post-intervention among adults following treatment. However, the effect at long follow-up and the improvement of function were not significant, and a considerable dropout rate was also found. Also, there was significant publication bias and other influential methodological problems including with self-rating, control condition and imputation. This may imply a probability of overestimation of the effect of CCBT and the need to further improve it. Nevertheless, it is possible that we do not need to be too pessimistic about CCBT, since we might be just midway on a long journey of low intensity intervention, of which CCBT is one part [67]. More careful research is required for CCBT to develop more substantially.

## Appendix: The search terms used and search process

MEDLINE, EMBASE

1. exp Depression/

2. exp Depressive Disorder/

3. (Depression or depressive or depressed).tw.

4. exp Dysthymic Disorder/

5. Dysthymia.mp.

6. exp Depressive Disorder, Major/

7. exp Affect/

8. exp Mental Disorders/

9. or/1-9

10. exp Internet/

11. exp Therapy, Computer-Assisted/

12. exp Cognitive Therapy/

13. exp Primary Health Care/

14. CCBT.mp.

15. exp Mental Health Services/

16. exp Software/

17. User manual.mp.

18. exp Internet/

19. exp Bibliotherapy/

20. Bibliotherapy.tw.

21. exp Cognitive Therapy/

22. Self-help.tw.

23. Medical informatics computing.mp.

24. exp Medical Informatics Computing/

25. exp Multimedia/

26. exp Computer-Assisted Instruction/

27. Computer assisted instruction.mp.

28. exp Decision Making, Computer-Assisted/

29. Computer-assisted.tw.

30. Computer&.tw.

31. Internet.tw.

32. Therapy, computer assisted.tw.

33. Web-based.mp.

34. Web-based.tw.

35. Beating the blues.mp.

36. Beating the blues.tw.

37. Overcoming depression.tw.

38. or/10-37

39. exp Psychotherapy/

40. psychotherapy.mp.

41. exp Psychotherapy, Multiple/

42. exp Psychotherapy, Group/

43. exp Psychotherapy, Brief/

44. (Cognitive adj2 therap$).tw.

45. ((Behaviour& or behavior&) adj2 therap&).tw.

46. Cognitive Therapy/

47. CBT.mp.

48. exp Psychoanalysis/

49. psychoanalysis.mp.

50. TAU.tw.

51. Treatment as usual.tw.

52. Standard treatment.tw.

53. Medication.tw.

54. exp Drug Therapy/

55. Pharmacotherapy.mp

56. Control.tw.

57. Supportive therapy.mp.

58. Supportive therapy.tw.

59. exp Waiting Lists/

60. Waiting list$.tw.

61. exp Placebos/

62. placebo.tw.

63. or/39-63

64. exp Randomized Controlled Trial/

65. Randomized controlled trial.mp.

66. Randomized controlled trial$.tw.

67. exp Randomized Controlled Trials as Topic/

68. RCT.mp.

69. RCT&.tw.

70. Random Allocation.tw.

71. or/64-71

72. 9 and 38 and 63 and 71

PsychINFO

1. exp "Depression (Emotion)"/

2. exp Major Depression/

3. Depression.mp.

4. (Depression or depressive or depressed).af.

5. dysthymia.mp. or exp Dysthymic Disorder/

6. or/1-5

7. Internet.mp. or exp Internet/or exp Internet Usage/

8. exp Cognitive Therapy/

9. exp Computer Applications/

10. exp Computers/

11. exp Computer Assisted Therapy/

12. exp Computer Assisted Instruction/

13. Therapy, Computer-Assisted.mp.

14. exp Computer Software

15. Computer assisted.af.

16. exp Computer Assisted Therapy/

17. Ccbt.mp.

18. exp Self Help Techniques/

19. ccbt.af.

20. Bibliotherapy.mp. or exp Bibliotherapy/

21. exp Online Therapy/or self-help.mp.

22. Self-help.af.

23. Web-based.mp.

24. Beating the blues.af.

25. or/7-24

26. exp Brief Psychotherapy/

27. exp Group Psychotherapy/

28. exp Psychotherapy/

29. exp Analytical Psychotherapy/

30. exp Individual Psychotherapy/

31. exp Interpersonal Psychotherapy/

32. exp Psychodynamic Psychotherapy/

33. tau.af.

34. treatment as usual.af.

35. standard treatment.af.

36. supportive therapy.af.

37. psychotherapy.af.

38. medication.mp. or exp Drug Therapy/

39. Pharmacotherapy.mp.

40. antidepressant$.tw.

41. Control&.af.

42. Waiting list$.af.

43. placebo.af.

44. or/26-44

45. exp Clinical Trials/or exp Intervention/or randomized controlled trial.mp.

46. randomised controlled trial$.af.

47. rct.mp.

48. random allocation.af.

49. or/45-49

50. (Cognitive adj2 therap$).af.

51. ((Behaviour& or behavior&) adj2 therap&).af.

52. Cognitive Therapy.mp.

53. exp Cognitive Therapy/

54. exp Cognitive Therapy/

55. exp Cognitive Behavior Therapy/

56. cbt.mp.

57. or/50-56

58. 6 and 25 and 44 and 49 and 57

## Competing interests

The authors declare that they have no competing interests.

## Authors’ contributions

MS worked on the whole of this study throughout the process of conceptualising and planning this research, conducting and selecting data, analysing the results and finally writing this paper as a corresponding author. SY carried out significant fundamental work, contributing to the design of the study, data collection and selection. SH also carried out significant work mainly in the process of data collection and selection. TF made a considerable contribution mainly to parts of advanced statistical analyses such as meta-analysing and processing publication bias. PM contributed to the whole work from initial idea generation through designing the protocol to checking the final version as MS’s supervisor. All authors read and approved the final manuscript.

## Authors’ information

MS MD, PhD is a visiting researcher of King’s College London Institute of Psychiatry whose specialty is modern psychotherapies including CBT or interpersonal psychotherapy, and who is engaged as a supervisor of CBT trainees in Japan. SY PSW PhD is a flexible researcher at the National Institute of Mental Health, National Center of Neurology and Psychiatry (NCNP) in Japan. His specialty is psychiatric rehabilitation and thus he is highly interested in low-intensity psychological intervention, including the usefulness of CCBT and bibliotherapy. SH MA is a lecturer at the Department of Psychology and Humanities, Edogawa University College of Sociology. Within his research, he has been engaged in self-help intervention for Japanese employees. Mitsuhiro Sado MD MSc is an instructor at Keio University School of Medicine whose major area of interest is the economic evaluation of psychological interventions such as CBT. He has a significant interest in CCBT, especially from the perspective of cost-effectiveness. TF MD PhD is a professor at Kyoto University Graduate School of Medicine/School of Public Health. He is not only a leading CBT supervisor in Japan but also a prominent epidemiologist in the country. PM PhD is a professor at the Centre for the Economics of Mental and Physical Health (CEMPH) at the Institute of Psychiatry King's College London. His specialty is the economic evaluation of mental health, and he has actually evaluated the cost-effectiveness of CCBT in the UK. http://www.iop.kcl.ac.uk/staff/profile/default.aspx?go=10869

## Pre-publication history

The pre-publication history for this paper can be accessed here:

http://www.biomedcentral.com/1471-244X/13/113/prepub
